# The MADS-box protein SHATTERPROOF 2 regulates *TAA1* expression in the gynoecium valve margins

**DOI:** 10.1007/s00497-024-00518-6

**Published:** 2025-01-10

**Authors:** Subodh Verma, Lenka Švihlová, Hélène S. Robert

**Affiliations:** Hormonal Crosstalk in Plant Development, Mendel Center for Plant Genomics and Proteomics, CEITEC MU—Central European Institute of Technology, Masaryk University, 625 00 Brno, Czech Republic

**Keywords:** SHATTERPROOF 2, YUCCA 4, TAA1, Valve margins, Gynoecium, Auxin

## Abstract

**Key message:**

SHATTERPROOF 2 regulates* TAA1* expression for the establishment of the gynoecium valve margins.

**Abstract:**

Gynoecium development and patterning play a crucial role in determining the ultimate structure of the fruit and, thus, seed production. The MADS-box transcription factor SHATTERPROOF 2 (SHP2) contributes to valve margin differentiation and plays a major role in fruit dehiscence and seed dispersal. Despite the acknowledged contribution of auxin to gynoecium development, its precise role in valve margin establishment remains somewhat enigmatic. Our study addresses this gap by uncovering the role of SHP2 as a positive regulator of key auxin biosynthetic genes, *TAA1* and *YUCCA 4*. Genetic and molecular analyses revealed that SHP2 directly regulates the expression of *TAA1* in the valve margins of a stage 12 gynoecium with known regulators of flower and ovule development, such as AGAMOUS, SEEDSTICK, and SEPATALA 3. Collectively, our findings define a previously unrecognized function of SHP2 in the regulation of auxin biosynthetic genes during gynoecium development and raise the possibility that the auxin produced under SHP2 regulation may contribute significantly to the valve margin establishment.

**Supplementary Information:**

The online version contains supplementary material available at 10.1007/s00497-024-00518-6.

## Introduction

In angiosperms, reproductive success relies on the coordinated development of the gynoecium, the female reproductive organ, and the anther, the male reproductive organ (Roeder & Yanofsky 2006). After fertilization, the gynoecium develops into the fruit, which protects the developing seeds until they are dispersed into the environment. This requires a better understanding of gynoecium development and patterning. In *Arabidopsis*, the gynoecium (or pistil) is formed by the congenital fusion of two carpels (Ramos-Pulido & de Folter 2023; Roeder & Yanofsky 2006). In the medial domain, at the margins of the fused carpels, the carpel margin meristem (CMM) develops and gives rise to the marginal tissues of the gynoecium, including the ovules, while in the lateral domains, valves and valve margins develop (Ramos-Pulido & de Folter 2023). By stage 11/12, the gynoecium encompasses all the modules for proper fruit patterning (Chávez Montes et al. 2015; Smyth et al. 1990). Following fertilization, the fruit and seeds begin to develop. As the fruit matures, the valve margin differentiates into the separation and lignified layers (Liljegren et al. 2000, 2004). These specialized cell layers ultimately contribute to fruit dehiscence and seed release (pod shattering). Controlling fruit development and seed dispersal is of great biological and economic importance. This can be achieved by understanding the molecular and genetic mechanisms behind gynoecium development, including the establishment and specification of the valve margin.

Auxin is involved in many aspects of plant growth and development, including gynoecium patterning and ovule development (Barro-Trastoy et al. 2020; de Folter 2016; Robert et al. 2015). Auxin enables proper organ polarity through its concentration/signaling gradients, maintained by local auxin production and inter- and intracellular transport. However, very few studies have been conducted to elucidate how local auxin biosynthesis is controlled during gynoecium development (Reyes-Olalde et al. 2017). In plants, indole-3-acetic acid (IAA) is the predominant natural auxin, synthesized mainly via tryptophan-dependent biosynthetic pathways (Mashiguchi et al. 2011). One of these pathways involves two steps: the conversion of tryptophan to indole-3-pyruvate (IPyA) by the TRYPTOPHAN AMINOTRANSFERASE OF ARABIDOPSIS 1 (TAA1) and the oxidative decarboxylation of IPyA by the YUCCA (YUC) family to produce IAA. During gynoecium development, these auxin biosynthetic genes exhibit distinct expression patterns in various locations, suggesting their involvement in the gynoecium morphogenesis. For instance, TAA1 is required for proper gynoecium development (Stepanova et al. 2008). The *TAA1* expression was reported in the medial domain of the stage 8–9 gynoecium. *YUC4* is expressed in the style of the mature gynoecium (Cheng et al. 2006). However, the molecular mechanisms regulating auxin biosynthesis and its precise contribution to gynoecium patterning remain unclear.

Gynoecium development and patterning are under the tight control of several transcription factors. Two closely related genes, *SHATTERPROOF 1* (*SHP1*) and *SHP2*, which encode MADS-box transcription factors AGL1 and AGL5, are required for valve margin differentiation (Liljegren et al. 2000). These two proteins act together with another MADS-box transcription factor, SEEDSTICK (STK), to control ovule identity (Pinyopich et al. 2003). A bHLH transcription factor, INDEHISCENT (IND), is another contributor to valve margin specification that acts downstream of the SHP proteins (Liljegren et al. 2004). However, its activity does not depend solely on SHP proteins. The expression of *SHP1/2* and *IND* is negatively regulated by FRUITFULL (FUL) in valve margin cells (Ferrándiz et al. 2000; Liljegren et al. 2004). It has been shown that IND helps establish an auxin minimum at the valve margin by modulating the localization of the auxin transporter PIN3. This auxin minimum is required for the specification of the valve margin separation layer in stage 17-B silique (Sorefan et al. 2009). In contrast, van Gelderen et al. (2016) reported that at stage 14, an increase in auxin levels is required for dehiscence zone formation in the valve margin. However, understanding the precise mechanisms of auxin transport and the maintenance of auxin gradients during gynoecium patterning is challenging without knowing how auxin production is regulated.

In the present study, we investigated a novel role for SHP2 in regulating the expression pattern of the auxin biosynthetic genes *TAA1* and *YUC4* during gynoecium development. Our molecular and genetic evidence suggests that SHP2 directly regulates the expression of *TAA1* in the valve margin. Furthermore, a multimeric complex containing SHP2, and its associated proteins has been identified.

## Materials and methods

### Plant lines and cultivation

All Arabidopsis mutant plants used in this study were in Columbia (Col-0) background. Seeds of *shp1 shp2* were ordered from NASC (CS3844). Seeds of *stk-2* were kindly provided by L. Colombo (Department of Biosiences, University of Milan) (Pinyopich et al. 2003). Plants homozygous for *shp1* and *shp2* alleles were detected as described previously (Liljegren et al. 2000). The *pTAA1:GFP-TAA1* and *pYUC4:3nxGFP* were previously described (Robert et al. 2018; Stepanova et al. 2008). Seeds were sterilized with chlorine gas and stratified at 4 °C for 24 h. Seeds were germinated on 1/2 MS plates containing selection antibiotics and grown at 21 °C under a long-day photoperiod. The primers used in the study are listed in Table S1. Two-week-old seedlings were transferred to soil (mixture of 2/3 peat moss Substrate 3 [Klasmann-Deilmann GmbH, Germany] and 1/3 vermiculite). Plants were grown in a walk-in Fytoscope growth chamber (FS-WI, Plant Systems Instruments (PSI), Czech Republic) under growth conditions with a long-day regime (16 h light/8 h dark), LED illumination with an intensity of 150 μmol m^−2^ s^−1^, and 35–45% humidity.

### Plasmid construction and plant transformation

*pSHP2:SHP2-GFP* and *pSHP2:GUS* were constructed using modular cloning (MoClo) (Weber et al. 2011). ~ 2 kb promoter sequence of *SHP2* was amplified using genomic DNA from Col-0 and cloned into the level 0 destination vector pICH41295. The coding sequence of *SHP2* was PCR-amplified using cDNA synthesized from the inflorescence. The amplified product was cloned into the level 0 destination vector pAGM1287. The generated level 0 modules were assembled with compatible sets of level 0 modules, such as GFP (pICSL50008), GUS (pICH75111) and terminator (pICH41414), into a level 1 destination vector, pICH47742. Another level 1 module was constructed for plant selection, containing phosphinotricin (PPT) by transferring pICSL70005 in pICH47802 destination vector. These level 1 modules were assembled into a level 2 destination vector, pAGM4673, to generate a level 2 module, which was transformed into the GV3101 Agrobacterium by electroporation. Transgenic plants were obtained by floral dip method (Zhang et al. 2006). Transformed plants were selected on 1/2 MS media supplemented with PPT. Further confirmation of transgenic plants was carried out by PCR genotyping using forward primer from GFP reporter and reverse primer from terminator (forward primer: ACGGCAACTACAAGACCCG; reverse primer: TCCGCTCACAATTCCACACA). Analyses were performed on T3 hemi- and homozygous plants.

### Dual luciferase assay

The promoter regions of *TAA1* and *YUC4* were cloned in front of the firefly luciferase in the pGreenII0800-LUC vector to generate the reporter constructs (*pTAA1:LUC and pYUC4: LUC)* with Renilla (REN) luciferase under the *35S* promoter as an internal control. To generate the effector constructs, the coding sequences of *SHP2* and *YFP* were cloned under the control of a *35S* promoter using modular cloning. *35S:YFP* was used as a negative control. Both effector and reporter plasmids were transformed into GV3101 and co-infiltrated into the leaves of 25–28-day-old *N. benthamiana* through Agrobacterium-mediated infiltration. The transformed plants were kept in the dark overnight and moved to the cultivation chamber with a long-day photoperiod. Luc/Ren ratio was measured 48 h after infiltration using the Dual-luciferase reporter kit (Promega) following the manufacturer’s instructions in the Promega GloMax Multiplus Plate Reader.

### RNA isolation and RT-qPCR

Total RNA was isolated from stage 12 gynoecium using a Qiagen RNA extraction kit. After DNase treatment (Promega), cDNA synthesis was carried out using an iScript cDNA synthesis kit per the manufacturer’s instructions. Quantitative PCR was conducted in LightCycler 96 (Roche) real-time detection system using FastStart Essential DNA Green Master mix (Roche). The constitutive *UBIQUITIN* gene was used as the endogenous control. The following PCR conditions were used: preincubation at 95 °C for 600 s, then 45 cycles of 95 °C for 10 s, 60 °C for 10 s, 72 °C for 20 s, followed by one cycle of melting at 95 °C for 10 s, 65 °C for 60 s and 97 °C for 1 s. Relative quantification (RQ) values were calculated using the 2 − ΔΔCt method (Livak & Schmittgen 2001). The RQ values in the mutant are normalized to the RQ values of Col-0. RQ values for Col-0 are set at 1, as previously published (Chakraborty et al. 2024).

### Immunoprecipitation followed by mass spectrometry

Total protein was isolated from 1 g of inflorescence tissue from Col-0 and *pSHP2:SHP2-GFP* plants. The samples were suspended in 3 mL of lysis buffer (50 mM Tris/HCl pH 7.5, 150 mM NaCl, 10 mM MgCl2, 10% glycerol, 1 mM EDTA, 2 mM DTT, 1% Triton X-100, NP40, 1 mM PMSF, 1 × protease inhibitor cocktail [Roche]) on ice. Immunoprecipitation (IP) was carried out using GFP-trap magnetic beads from ChromoTek following the manufacturer`s instructions. Col-0 wild-type plants were used as a control. The control plants underwent the same processing as the GFP-tagged plants. Three independent biological replicates for each sample were processed and analysed.

LC–MS/MS measurements were performed on a timsTOF Pro mass spectrometer (Bruker Daltonics) hyphenated with a Dionex UltiMate^™^ 3000 RSLCnano system (Thermo Scientific). DIA LC–MS data processing is done using the DIA-NN application (version 1.8; https://github.com/vdemichev/DiaNN).

### Chromatin immunoprecipitation and qPCR

Chromatin immunoprecipitation assay was carried out according to the described protocol (Gendrel et al. 2005) with minor modifications. To extract chromatin, ~ 1 g of inflorescence tissue from wild-type (Col-0) and *pSHP2:SHP2-GFP* plants was used. GFP trap agarose beads (Chromotek) were used to immunoprecipitated the chromatin. After the reversal of cross-linking, ChIP DNA was purified manually using phenol:chloroform:isoamyl alcohol (25:24:1, pH 8). The enrichment of the binding regions was analyzed using quantitative PCR using SYBER Green Supermix and LightCycler 96 (Roche) real-time detection system. The relative enrichment of the target regions obtained from *pSHP2:SHP2-GFP* was compared with the enrichment measured from the wild type. *ACTIN* 2/7 was used for normalization. Fold enrichment was calculated as previously described (Matias-Hernandez et al. 2010).

### Microscopy

Ovules with septum were collected from gynoecium, mounted in water, and observed under LSM780 confocal microscope. The stage 12 gynoecium was removed from the flower, fixed in 4% PFA, cleared in ClearSee alpha solution following the protocol mentioned in Attuluri et al. (2022), and observed under a Zeiss LSM 700 laser scanning confocal microscope. Seeds were collected at 4 days after pollination, fixed and cleared as described above (Attuluri et al., 2022). Observations were made with a 25 × magnification objective. GFP imaging was performed using a 488 nm laser. Images were analyzed with the ZEISS ZEN software. The Z-stack of the *pTAA1::GFP-TAA1* samples was projected on the orthogonal axis to reconstruct a Z-axis optical cross-section. The expression profile along a line perpendicular to the silique was performed on the surface section of a Z-stack with the ZEISS ZEN software and exported in Excel. The average of the fluorescence signal from 7 Col-0 and 5 *shp1 shp2* siliques was plotted along the transversal X-axis.

For scanning electron microscopy, freshly dissected gynoecium was visualized in a dual beam FIB/SEM Versa 3D microscope equipped with a Quorum Technologies PP3010T cryo-stage and cryo-transfer chamber. The gynoecium was dissected and mounted onto the aluminum electron microscopy stub with conductive (copper or carbon) adhesive tape, fixed into the sample holder, and rapidly frozen into liquid nitrogen. In the transfer chamber, samples were sublimed (180 °C, 10 min) and sputtered (5–6 × 10–2 mbar, 60 s) with platinum. The samples were then transferred to the microscope chamber (−191 °C, 2–3 × 10–5 Pa). The microscope chamber is equipped with Everhart–Thornley (ETD) detector, an ion conversion and electron (ICE) detector, and a retractable concentric back-scatter electron detector (CBS). Samples were imaged in a eucentric position (working distance = 10 mm). Other parameters, such as the high voltage and the magnification, varied between samples.

### Histology

The *shp1 shp2* and Col-0 gynoecium were fixed for 16 h at room temperature in 50% ethanol, 5% glacial acetic acid, and 3.7% formaldehyde and dehydrated through an ethanol dilution series. The tissues were embedded in paraffin and the basal part of the gynoecium was cut in 10 μm section with a RM 2125 rotary microtome (Leica). The slides were deparaffinated in 2 × 100 5 Histoclear, 2 × 100% ethanol, air-dried and stained with 0.05% Toluidine blue O (Sigma Aldrich) for structural evaluation. Lignin was detected with 0.5% Safranin-O (Acros organics) in an aqueous solution containing 0.3% Alcian blue (Sigma-Aldrich) followed by three washes in water. Lignin was also detected by staining with 2% phloroglucinol (Duchefa) solution in 96% ethanol for 2 min followed by 50% HCl for 30 s. Slides were mounted in Entellan (Sigma-Aldrich). The samples were visualized using a ZEISS AxioScope light microscope.

Gynoecium of *pSHP2:GUS* was stained for GUS according to a published protocol (Moubayidin & Ostergaard 2014), and cross-sections were prepared as described above. After removal of the paraffin, the slides were mounted in Entellan (Sigma Aldrich) and samples were visualized using a ZEISS AxioScope light microscope.

Figures were assembled in Microsoft PowerPoint.

## Results

### SHP2 has a dynamic expression pattern during gynoecium development

We generated *pSHP2:SHP2-GFP* transgenic lines to study the localization of the SHP2 protein during gynoecium development. The functionality of the translational fusion was assessed after crossing into the *shp1 shp2* genetic background. The *pSHP2:SHP2-GFP shp1 shp2* plants produced siliques in which the valve margin lignification was indistinguishable from the wild type, demonstrating that the SHP2-GFP fusion protein was biologically active and able to fully compensate for the absence of the endogenous SHP2 protein (Supplementary Fig. 1).

**Fig. 1:**
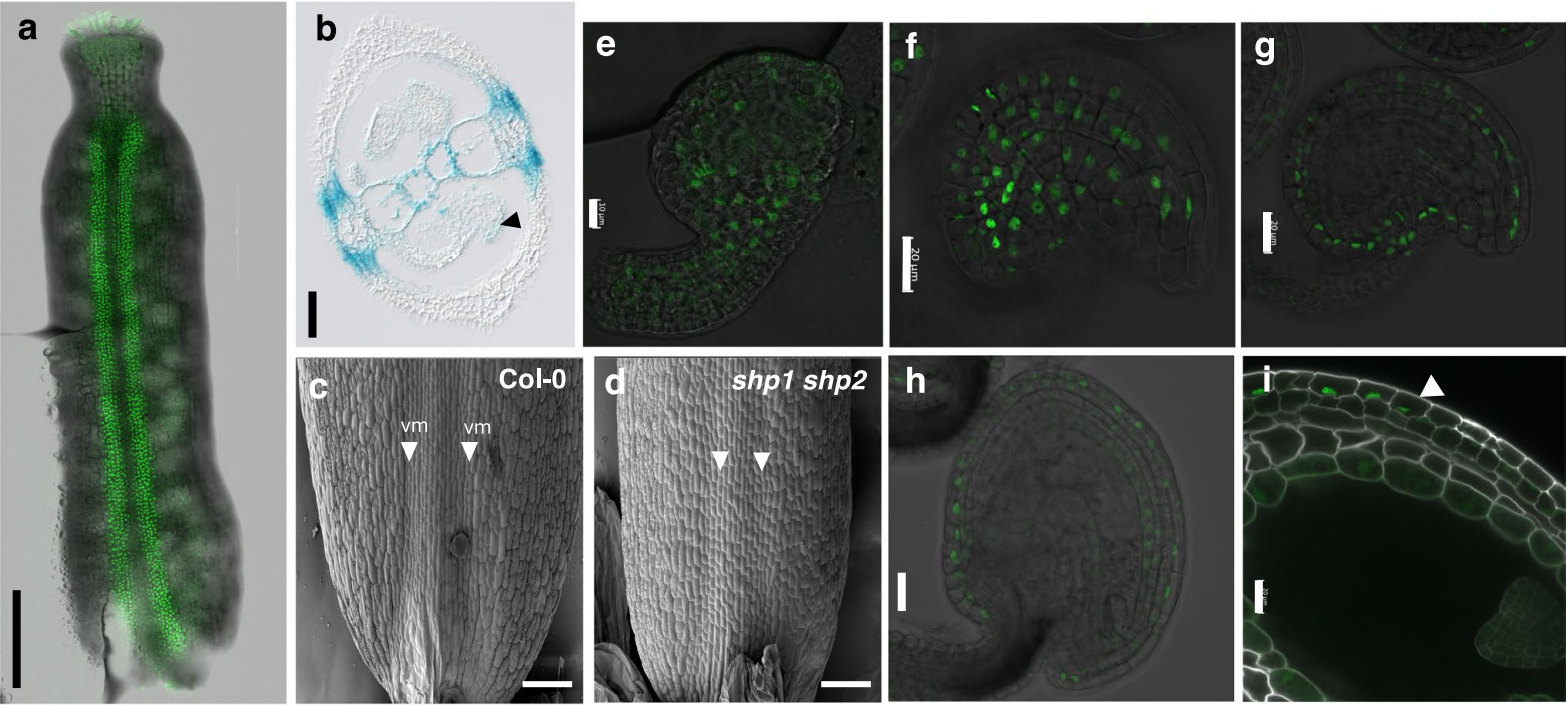
The expression of *SHP2* at the junction between valve and replum of the gynoecium is required for its specification **a**, **b** SHP2 is expressed at the valve-replum junction in stage 12 gynoecium. A gynoecium expressing SHP2-GFP **a** and a cross-section of a gynoecium expressing *pSHP2:GUS* **b** are presented. The black arrowhead marks SHP2 expression in the ovule integuments. **c**, **d** SEM images of wild type (Col-0) and *shp1 shp2* mutant gynoecium (stage 12). White arrowheads indicate the valve margin (vm). Stage of gynoecium is according to (Smyth et al. 1990). **e**–**g** In developing ovules, the expression is detected in the integuments. Young ovule (stage 3-I) **e**; late-stage ovule (3-V/3-VI) images taken at different focal distance **f**, **g**; post fertilization (stage 4-II/4-III) **h**. SHP2-GFP expression in the outermost layer of the seed coat at the heart stage of seed development **i**. Stages of ovule development are according to (Schneitz et al. 1995). The fluorescent signal is green. The white signal in **e** is the Renaissance dye counterstaining cell walls. The GUS signal is blue. Scale bars: 50 μm **a**, 100 μm **b**–**d**, 10 μm **e**, 20 μm **f**–**i**

We detected SHP2-GFP expression in the valve-replum junction (valve margin) in stage 12 gynoecium (Fig. 1a). Cells in this region later differentiate and lignify during fruit development. The sharp *SHP2* expression at the valve-replum junction was confirmed with a transcriptional fusion (*pSHP2:GUS*) on transversal sections of the gynoecium (Fig. 1b). Consistent with this expression pattern, we observed morphological differences in the valve margin by scanning electron microscopy between wild-type and *shp1 shp2* pistils at stage 12 (Fig. 1c, d). The valve margin was less defined in *shp1 shp2* gynoecium compared to wild-type gynoecium. However, these differences were not visible on transversal sections of the gynoecium (Supplementary Fig. S2). This indicates that SHP2 is required to specify the valve margin region before fertilization at stage 12. SHP2-GFP was also detected in the integuments of the developing ovules (Fig. 1e–g), also visible in *pSHP2:GUS* gynoecium after GUS staining (Fig. 1b). At stages (3-V to 4-VI), SHP2-GFP expression was strongly observed in the layers of the outer integument. During seed development up to the heart stage, the expression was restricted to the outermost layer of the seed coat (Fig. 1h, i). Our observations of *SHP2* expression in pistils are consistent with previous reports (Sehra and Franks 2017), while the localization of SHP2 protein in ovules and seeds is novel.

### SHP2 regulates the expression of auxin biosynthetic genes during gynoecium development

**Fig. 2:**
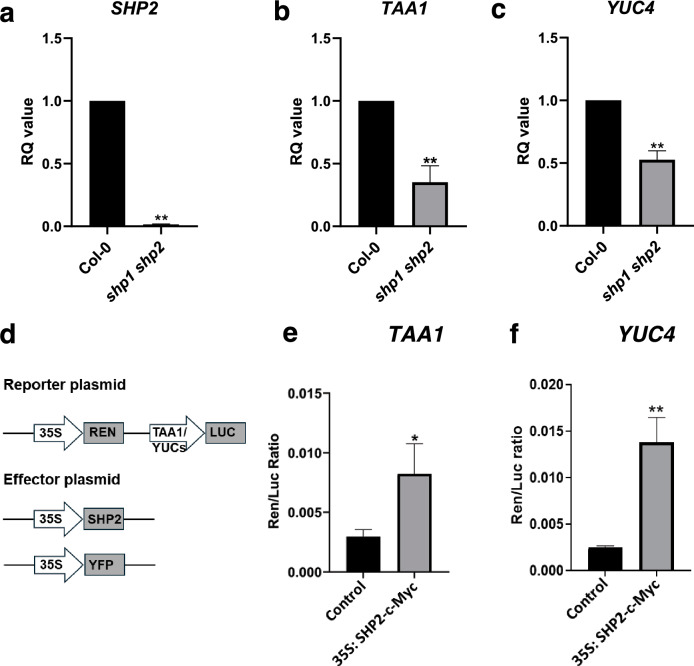
*TAA1* and *YUC4* expression is regulated by SHP2 **a**–**c** The expression of *SHP2* **a**, *TAA1* **b** and *YUC4* **c** is reduced in *shp1 shp2* gynoecium (stage 12). Relative quantification of transcript levels assessed by RT-qPCR in Col-0 and *shp1 shp2* gynoecium. **d** Schematic of reporter and effector constructs used in the dual luciferase assays. **e**, **f** Ren/Luc ratio for *TAA1* **e** and *YUC4* **f** promoters after co-infiltration with *35S:YFP* (control) and *35S:SHP2-cMyc*. RENILLA (REN) was used as an internal control. The values are represented as the means ± SD from four biological replicates. *p*-values are calculated with a two-tailed Student’s *t*-test. *P < 0.05; **P < 0.01

In the publicly available transcriptomes performed on gynoecium tissues, *SHP2* was found to be co-expressed with *TAA1* and *YUC4* in the medial domain of the gynoecium (Luna-García et al. 2024; Villarino et al. 2016). To test the hypothesis that SHP2 could regulate the expression of *TAA1* and *YUC4*, we analyzed their transcript levels in stage 12 *shp1 shp2* gynoecium using RT-qPCR. Like *SHP2*, transcripts of *TAA1* and *YUC4* were significantly lower in the *shp1 shp2* mutant than in the wild type (Fig. 2a–c). We further conducted a transient dual luciferase assay in *N. benthamiana* to assess the regulatory effect of SHP2 on the transcriptional activity of the *TAA1* and *YUC4* promoters. We observed that SHP2 significantly enhanced the expression of *LUCIFERASE* under the control of *YUC4* and *TAA1* promoters, indicating that SHP2 can ectopically activate the transcription of *TAA1* and *YUC4* (Fig. 2d–f).

**Fig. 3:**
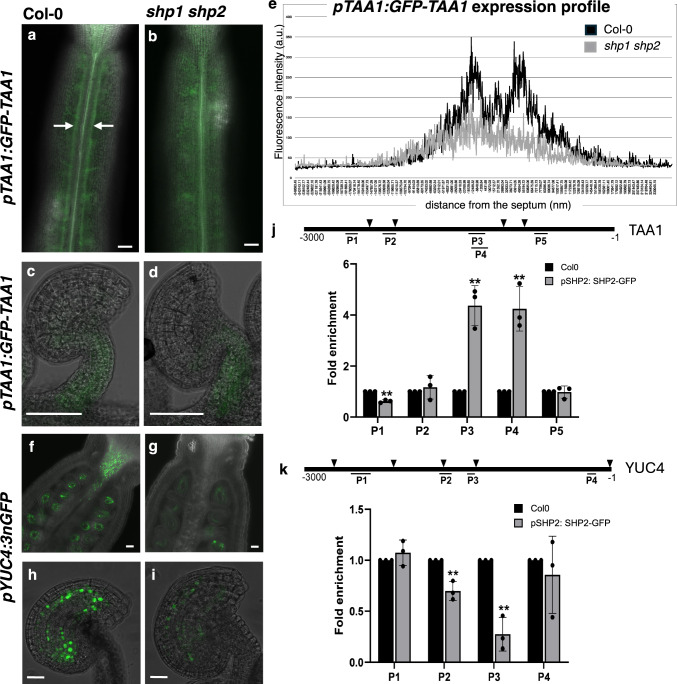
Interaction of SHP2 on *TAA1* promoter regulates its expression in gynoecium **a**, **b** *pTAA1:GFP-TAA1* expression at the valve-replum junction (white arrow) in wild-type gynoecium (stage 12) **a** is absent in *shp1 shp2* gynoecium **b**. The signal at the silique surface is presented. The full Z-stacks are presented in supplementary videos S1 and S2. A close-up of TAA1 expression in wild-type is presented in Supplementary Fig. S3. **c**, **d** *pTAA1:GFP-TAA1* expression in 3-V/3-VI ovules from stage 12 gynoecium of Col-0 **c** and *shp1 shp2* **d**. **e** GFP-TAA1 expression profile (fluorescence intensity, arbitrary unit (a.u.)) along the X-axis (in nm) on the surface of Col-0 (black) and *shp1 shp2* (grey) siliques (n = 7 for Col-0, n = 5 for *shp1 shp2*). **f**–**i** *pYUC4:3xnGFP* is expressed in the style of the silique in Col-0 **f** but not in *shp1 shp2* **g**. The full Z-stacks are presented in supplementary videos S3 and S4. *pYUC4:3xnGFP* signal is visible in the ovule inner integuments in wild type **h**. *YUC4* expression is reduced in *shp1 shp2* ovules **i**. The fluorescent signal is green **a**–**d**, **f**–**i**. The Renaissance dye stained the cell membranes **a**, **b**, **f**, **g**. Scale bars: 50 μm **a**–**d**, **f**, **g**, 20 μm h, **i**. **j** Schematic representation (top) of *TAA1* promoter showing the positions of CArG boxes (solid triangle) within the 3 kb region upstream of ATG. P1-5 indicates the location of amplicons used for ChIP-qPCR analysis. The result of ChIP-qPCR (bottom) shows the enrichment of regions P3 and P4 in the SHP2-GFP IP sample. **k** Schematic representation of *YUC4* promoter (top) showing the positions of CArG boxes (solid triangle) within the 3 kb region upstream of ATG. P1-4 indicates the location of amplicons used for ChIP-qPCR analysis. The result of ChIP-qPCR (bottom) shows a reduced level of P2 and P3 amplicons in the SHP2-GFP IP sample. The values are represented as the means ± SD from three independent experiments. *p*-values are calculated with a two-tailed Student’s *t*-test. *, *p* < 0.05; **, *p* < 0.01

In another approach, we analyzed the spatial expression pattern of *TAA1* and *YUC4* in the *shp1 shp2* mutant background using *pTAA1*:*GFP*-*TAA1* and *pYUC4*:*3xnGFP* lines, respectively. In the wild type, the GFP-TAA1 fluorescent signal was detected in the valve margin region of the stage 12 gynoecium (Fig. 3a; Supplementary Fig. S3; Supplementary Video S1). In contrast, it was notably absent in the same region in the *shp1 shp2* double mutant (Fig. 3b; Supplementary Video S2). This was quantitatively assessed by comparing the GFP-TAA1 fluorescence signal profile at the silique surface in *pTAA1*:*GFP*-*TAA1* and *pTAA1*:*GFP*-*TAA1 shp1 shp2*, which showed a decrease in signal intensity in the middle region of the silique in the mutant compared to Col-0 (Fig. 3e). Moreover, the GFP-TAA1 fluorescent signal was observed in the funiculus of the ovules. This expression remained unchanged in *shp1 shp2* ovules (Fig. 3c, d). It suggests that SHP2 is required for *TAA1* expression in the valve margin of the stage 12 gynoecium. *YUC4* was not expressed in the valve margins in Col-0 and *shp1 shp2* siliques (Supplementary Videos S3 and S4). However, in the wild type, *YUC4* expression was detected in the style and in the ovule integuments and embryo sac (Fig. 3f, h). In the *shp1 shp2* siliques, *YUC4* expression was absent in the style, while its intensity was notably reduced in the *shp1 shp2* ovules (Fig. 3g, i).

### SHP2 directly regulates TAA1 expression

To determine whether SHP2 directly regulates the expression of *TAA1* and *YUC4 *in vivo, we performed a chromatin immunoprecipitation (ChIP) assay followed by qPCR. We screened 3 kb promoter sequences of *TAA1* and *YUC4* for the presence of MADS-box transcription factor binding sites (CArG). The promoter region of *TAA1* contains 4 CArG motifs, whereas the *YUC4* promoter sequence contains 5 CArG motifs (Fig. 3j, k). Among the four regions tested for binding of SHP2, the ChIP-qPCR revealed a significant enrichment of the region spanning the CArG boxes 3 and 4 of the *TAA1* promoter in *pSHP2*:*SHP2-GFP* inflorescence compared to the wild-type inflorescence (Fig. 3j). However, despite the presence of 5 CArG motifs in its promoter, no binding of SHP2 to the *YUC4* promoter was observed by ChIP-qPCR assay (Fig. 3k). This suggests that SHP2 binds to the *TAA1* promoter to activate its expression, whereas SHP2 indirectly regulates the expression of *YUC4*.

### SHP2 predominantly interacts with MADS-box proteins in inflorescence

To further explore the molecular mechanism of SHP2 and to systematically identify its interacting partners, we immunoprecipitated the protein complex from *pSHP2*:*SHP2-GFP* inflorescence using antibodies against GFP and characterized it by LC–MS/MS. Fifteen proteins were enriched in the IP samples compared to control samples (Table 1; Table S2). The most significantly enriched protein was SHP2, confirming the specificity of the IP protocol. The other proteins with the highest confidence enrichment in the IP fraction were AGL18, AGL15, and SHP1. Of the 15 proteins, ten belonged to the MADS-box protein family. In addition to MADS-box proteins, several other proteins, such as PPIase FKBP53, probable ubiquitin-like specific protease 2B, and uncharacterized protein F9D24.20, were enriched in the IP samples. GO enrichment analysis revealed that 10 out of 15 proteins were nuclear localized and associated with DNA binding activity (Table S2). Proteins known to play key roles in flower/gynoecium and ovule development, such as AGAMOUS (AG), STK, and SEPALLATA 3 (SEP3), were also enriched.

**Table 1:** Proteins binding the SHP2-GFP in inflorescence

Sample	Description	log FC	*p*-value
1	SEPALLATA 3 (Agamous-like MADS-box protein AGL9)	5.60	4.93E−06
2	MADS-box protein AGL5 (Protein SHATTERPROOF 2)	14.29	1.62E−05
3	Peptidyl-prolyl cis–trans isomerase FKBP53 (PPIase FKBP53)	6.35	1.2E−05
4	MADS-box protein AGL18	12.96	3.74E−08
5	Probable ubiquitin-like-specific protease 2B (EC 3.4.22.-)	4.73	5.79E−05
6	Uncharacterized protein F9D24.20	4.47	4.23E−05
7	Floral homeotic protein AGAMOUS	2.91	0.000249
8	MADS-box protein AGL11 (Protein SEEDSTICK)	4.63	8.53E−05
9	Floral homeotic protein APETALA 3	3.00	8.98E−05
10	MADS-box protein AGL15	10.86	5.2E−08
11	At3g12510 (MADS-box family protein) (Uncharacterized protein At3g12510)	10.02	1.79E−06
12	K-box region and MADS-box transcription factor family protein	8.69	1.75E−06
13	Hypothetical Ser-Thr protein kinase (Uncharacterized protein At2g40120)	7.56	2.66E−07
14	AT5G19900 protein (PRLI-interacting factor)	6.97	2.61E−06
15	MADS-box protein SHORT VEGETATIVE PHASE	6.56	7.7E−06

*FC fold change

*STK* and *SHP2* are both expressed in the integuments of the ovule (Fig. 1e–g) (Mizzotti et al. 2014). While *SHP2* is restricted to the outer integuments, *STK* is present in all integument layers (Mizzotti et al. 2014), including the inner integument layer where *YUC4* is expressed (Fig. 3h). *STK* is also expressed in the funiculus (Mizzotti et al. 2014), where *TAA1* is present (Fig. 3c). In addition, STK is required for funiculus development and is redundant with SHP1 and SHP2 to promote ovule identity (Pinyopich et al. 2003; Favaro et al. 2003). Since *YUC4* expression is reduced in *shp1 shp2* (Figs. 2c, 3i), but SHP2 does not bind to the CArG motifs on the *YUC4* promoter (Fig. 3l), we tested whether STK could be the MADS-box factor regulating *YUC4* expression in ovules. However, no significant changes in *TAA1* and *YUC4* expression levels were observed in *stk* pistils (Supplementary Fig. S4). These results are consistent with the transcriptomes of *stk* inflorescences, where no misregulated auxin biosynthetic genes were found (Mizzotti et al. 2014).

## Discussion

Auxin plays an essential role in gynoecium development and patterning. Such developmental events rely on the establishment of auxin maxima and minima, a process sustained by local auxin biosynthesis coupled with inter- and intracellular transport. Despite this understanding, the precise function of auxin biosynthesis and its transcriptional regulation during gynoecium development remains unclear. In this study, we examined stage 12 gynoecia and observed that the expression of the auxin biosynthetic genes *TAA1* and *YUC4* is regulated by SHP2 in the valve margin and ovules, respectively.

SHP2 and its homolog, SHP1, are required for valve margin differentiation, which occurs after fertilization (Liljegren et al. 2000). This pivotal developmental process facilitates pod shattering and seed dispersal (Roeder and Yanofsky 2006). However, even before fertilization, an outline of the valve-replum boundary becomes apparent in the stage 12 gynoecium. We observed the SHP2 localization in this region, suggesting its involvement in the establishment of the valve margin before fertilization. Previous studies have implicated auxin biosynthetic genes in gynoecium development and patterning. Stepanova et al. (2008) observed severe defects in the gynoecium of the *taa1* mutants, highlighting the importance of this gene for proper tissue patterning and development. Likewise, YUC4 is required for proper patterning and development of the gynoecium (Cheng et al. 2006). In a previous transcriptome analysis of *pSHP2:YFP*-expressing gynoecium tissue, *TAA1* and *YUC4* were observed to be co-expressed with *SHP2* in the medial domain of the gynoecium (Villarino et al. 2016). Similarly, in a transcriptome of the gynoecium, *TAA1* and *YUC4* were enriched in the medial domain samples (Luna-García et al. 2024), confirming the published expression pattern observed with reporter lines (Cheng et al. 2006; Stepanova et al. 2008). However, these studies focused primarily on early gynoecium tissues (stage 10) and did not include the valve margin region. In our study, we observed that the expression of *SHP2* and *TAA1* overlapped in the valve margin at stage 12. Interestingly, this valve margin expression was absent in the *shp1 shp2* mutant. Consistently, *TAA1* transcript levels were also reduced in the *shp1 shp2* mutant, strongly suggesting that SHP2 is required for *TAA1* expression in the valve margin. This finding aligns with a previous report where a similar expression pattern of *TAA1* in the valve margin at stage 12 was diminished in the *ngatha* mutant (Martínez-Fernández et al. 2014). Interestingly, we observed less *YUC4* promoter activity using a transcriptional reporter in *shp1 shp2* ovules and style, which was consistent with reduced *YUC4* transcript levels in *shp1 shp2* gynoecium detected by qPCR. However, the GFP fluorescence signal driven by the *YUC4* promoter was detected predominantly in the inner integument layers and embryo sac of the ovules and in the style, where the SHP2 protein is not expressed. Similarly, a previous report showed that *pSHP2*: GFP expression was absent in the style of stage 12 gynoecium (Sehra and Franks 2017). However, SHP2 expression was detectable in the style at later stages of gynoecium development through analysis of reporter expression driven by *pSHP2* (Colombo et al. 2010; Sehra and Franks 2017).

The presence of MADS-box binding CArG elements in the promoters of auxin biosynthetic genes suggests a possible interaction of MADS-box transcription factors. Indeed, we observed that SHP2 can activate the expression of *TAA1* and *YUC4* using a transient dual-luciferase assay, where SHP2 is overexpressed under the control of the *35S* promoter in *Nicotiana benthamiana* leaves. Using ChIP coupled with qPCR, we detected a direct binding between SHP2 and the *TAA1* promoter; however, no such binding was observed at the *YUC4* promoter. This suggests that the transcriptional regulation of *YUC4* by SHP2 in ovules is indirect.

According to a previous study, an auxin minimum is required for the valve margin specification (to specify the separation layer) at a late stage of fruit development (stage 17) (Sorefan et al. 2009). IND plays a crucial role in creating auxin minima by modulating PIN3 localization, thereby influencing the direction of auxin transport (Sorefan et al. 2009). At stages 14–16, the reduction of PIN3 abundance resulted in enhanced auxin levels in valve margin cells and affected valve margin differentiation (van Gelderen et al. 2016). While these reports have highlighted the importance of auxin levels in valve margin development and specification, it is essential to determine the specific site of auxin production during relevant stages of gynoecium development. Based on our current findings, we speculate that the auxin produced in the valve margin region of the gynoecium is transported to generate an auxin gradient that is used to establish the valve margin region.

MADS-box proteins are thought to function within multimeric complexes to regulate the expression of their target genes. In the current study, we conducted IP followed by mass spectrometry using *pSHP2*:*SHP2-GFP* to systematically identify its novel associated partners. In our analyses, SHP2 interacts predominantly with MADS-box proteins. In a previous study documenting the interacting proteins of STK, it was observed that STK, SHP1, SHP2 and AG form a complex in which they interact with each other through SEP3 (Favaro et al. 2003). Notably, all of these proteins were also identified in our SHP2-containing protein complex. This also leads us to speculate that SHP2 binding to the *YUC4* promoter may involve other proteins within the complex. We hypothesized that STK would be a good candidate, given its expression pattern in ovules (Mizzotti et al. 2014). However, neither *YUC4* nor *TAA1* expression was altered in *stk* pistils. Another such protein could be AG, as AG binds directly to the *YUC4* promoter and activates its expression in flower buds (Yamaguchi et al. 2018). One possibility is that SHP2 may regulate the expression of AG, which in turn controls the expression of *YUC4*. We observed that the *YUC4* expression is strongly reduced in the style of the *shp1shp2* gynoecium. Although the *shp1 shp2* style appears normal, the *crabs claw (crc) aintegumenta (ant) shp1 shp2* quadruple mutant exhibits severe defects in gynoecium patterning, including style development (Colombo et al. 2010). This suggests that SHP1 and SHP2 genetically interact with ANT and CRC to regulate style development. It has been shown that CRC binds to *YUC4* promoter and regulates its expression in floral bud (Yamaguchi et al. 2018). Thus, it is possible that the genetic interaction among SHP2, CRC and ANT play a crucial role in controlling *YUC4* expression in the style. Furthermore, it has been shown that STYLISH 1 (STY1) plays a key role in the normal development of the style and regulates the expression of *YUC4* (Sohlberg et al. 2006). Although SHP2 does not regulate STY1 expression in the gynoecium, a genetic relation between STY1 and SHP2 may exist (Colombo et al. 2010). Together, these findings raise the possibility that there are still unidentified factors between SHP2 and YUC4 that influence *YUC4* expression during gynoecium development. However, further experiments are needed to address these aspects of SHP2 function.

In addition to MADS-box proteins, other proteins such as FKBP (FK506 binding protein) type immunophilin, AtFKBP53, ubiquitin-like specific protease 2B, and uncharacterized protein F9D24.20 were enriched in the IP sample. AtFKBP53, a histone chaperone, plays a role in chromatin remodeling and regulates the expression of ribosomal DNA (Li & Luan 2010). According to a previous yeast two-hybrid study, the C2H2 zinc finger transcription factor NO TRANSMITTING TRACT (NTT) was found to physically interact with SHP2 (Marsch-Martínez et al. 2014). However, NTT was not detected in our SHP2-GFP IP experiment, likely due to the low abundance of this protein in the inflorescence tissues utilized in our analysis.

Taken together, we uncovered a novel role for SHP2 in regulating the expression of the auxin biosynthetic genes *TAA1* and *YUC4*. Our findings provide crucial insights into the mechanism of auxin production, essential for proper gynoecium development and patterning.

## Author contribution statement

SV and HSR conceived the project. SV and LŠ conducted experiments. SV and HSR analyzed data and wrote the manuscript. All authors read and approved the manuscript.

## Supplementary Information

Below is the link to the electronic supplementary material.

Supplementary file1 (PDF 2761 KB)

Supplementary file2 (XLSX 5486 KB)

Supplementary file3 (AVI 5299 KB)

Supplementary file4 (AVI 6938 KB)

Supplementary file5 (MOV 51849 KB)

Supplementary file6 (MOV 47883 KB)

Supplementary file7 (DOCX 15 KB)
