# Supplementary material for: The MADS-box protein SHATTERPROOF 2 regulates TAA1 expression in the gynoecium valve margins
Source: Plant Reprod. 2025 Jan 10;38(1):6. doi: 10.1007/s00497-024-00518-6 (PMC11723901; doi:10.1007/s00497-024-00518-6)
Supplement: Supplementary file 1 — Supplementary file1 (PDF 2761 KB) [file 497_2024_518_MOESM1_ESM.pdf]

Supplementary information

Plant Reproduction

The MADS-box protein SHATTERPROOF 2 regulates *TAA1* expression in the gynoecium valve margins

Subodh Verma, Lenka Švihlová, Hélène S. Robert

Hormonal Crosstalk in Plant Development, Mendel Center for Plant Genomics and Proteomics, CEITEC MU—Central European Institute of Technology, Masaryk University, 625 00 Brno, Czech Republic

Corresponding author: H. S. Robert, helene.robert.boisivon@ceitec.muni.cz

ORCID

SV 0000-0001-9071-037X

HSR 0000-0002-6610-836X

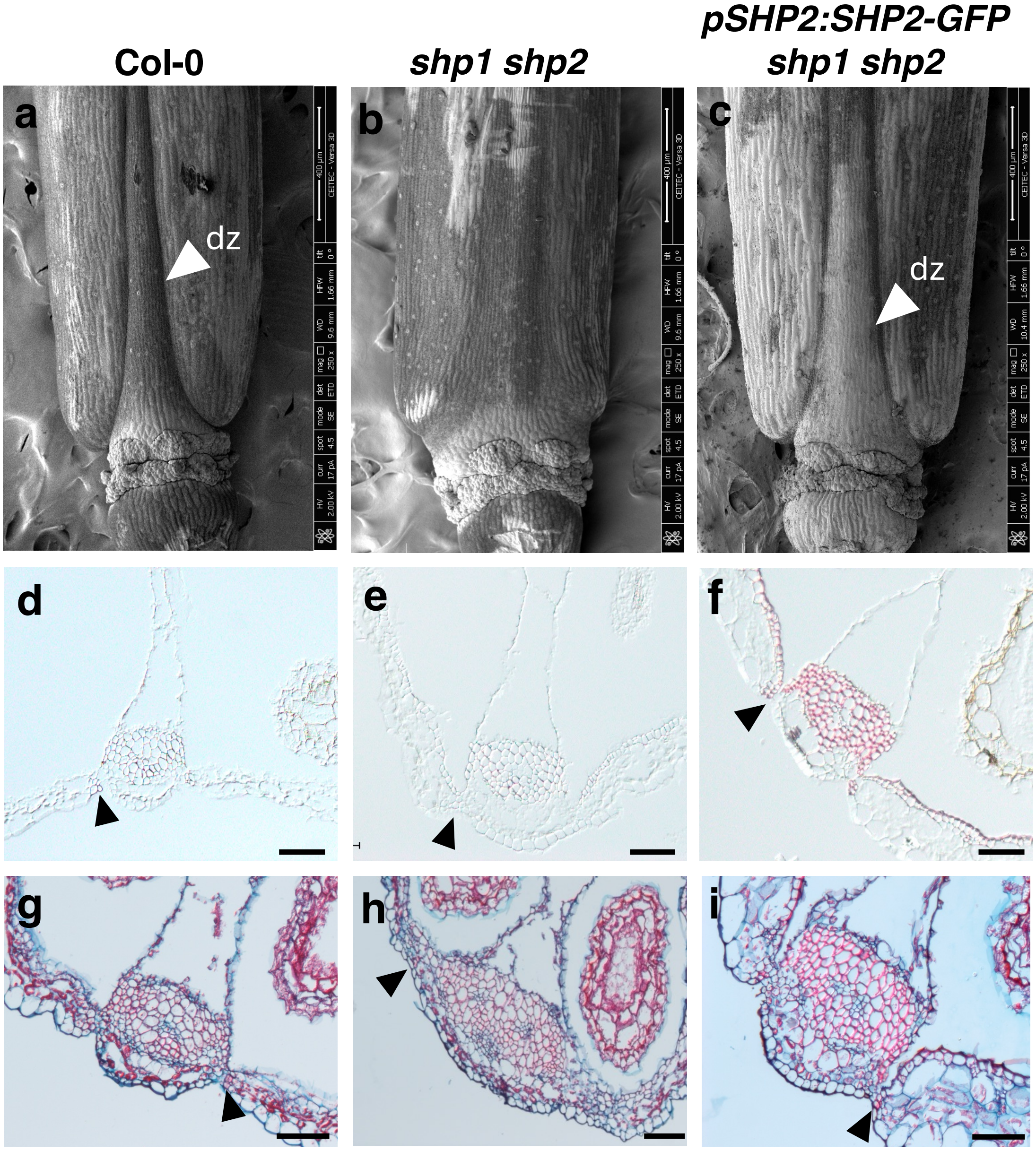

Supplementary Fig. S1 Complementation of the phenotype of *shp1 shp2* mutant by *pSHP2: SHP2-GFP*

The Col-0 siliques (stage 17b) exhibit a well-developed dehiscence zone (dz). The dehiscence zone is absent in *shp1 shp2* mutant (Liljegren et al., 2000). The presence of *pSHP2: SHP2-GFP* restores the *shp1 shp2* mutant phenotype. (**a-c**) The complementation was analyzed by SEM on siliques of the same genotypes. (**d-i**) Lignification of the valve margin was assessed with phloroglucinol staining (**d-f**) and Safranin-O combined with Alcian Blue (**g-i**) on transversal sections at the base of the silique. The lignified cells marked by the dye are indicated by a black arrowhead. The genotypes are Col-0 (a, d, g), *shp1 shp2* (b, e, h) and *pSHP2: SHP2-GFP shp1 shp2* (c, f, i). Scale bars 100 μm.

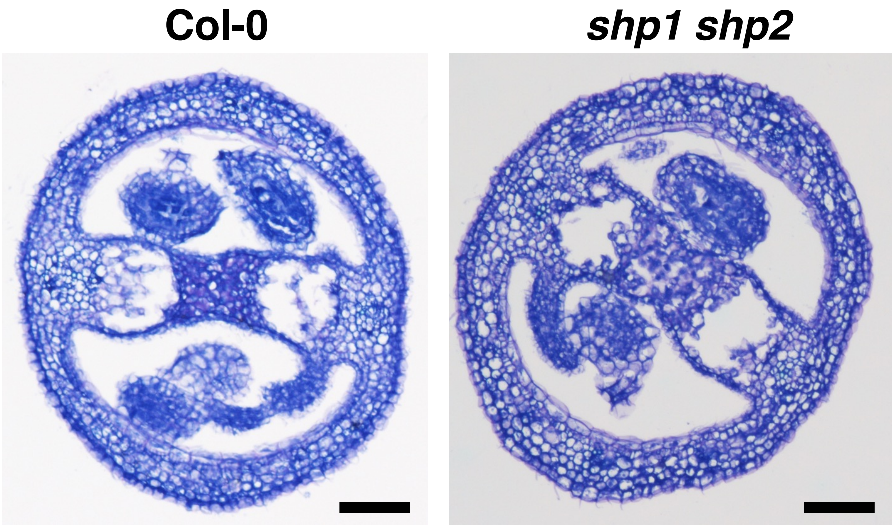

Supplementary Fig. S2 No obvious abaxial-adaxial defects in *shp1 shp2* gynoecium

Cross-section of wild-type and *shp1 shp2* stage 12 gynoecium. Cells are marked by Toluidine blue. Scale bars: 100 μm.

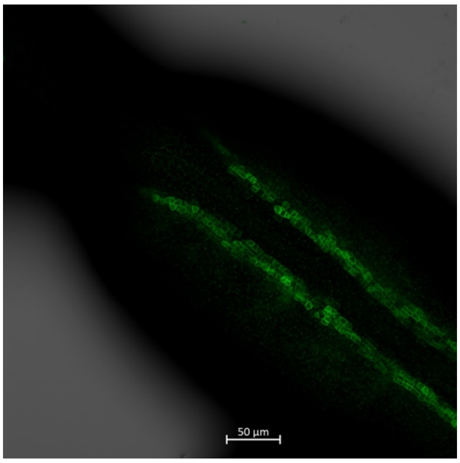

Supplementary Fig. S3 Close-up of *pTAA1:GFP-TAA1* expression pattern in stage 12 pistil

pTAA1:GFP-TAA1 expression in the cells at the valve-replum junction in wild-type gynoecium (stage 12). The fluorescent signal is green. Scale bar: 50 µm.

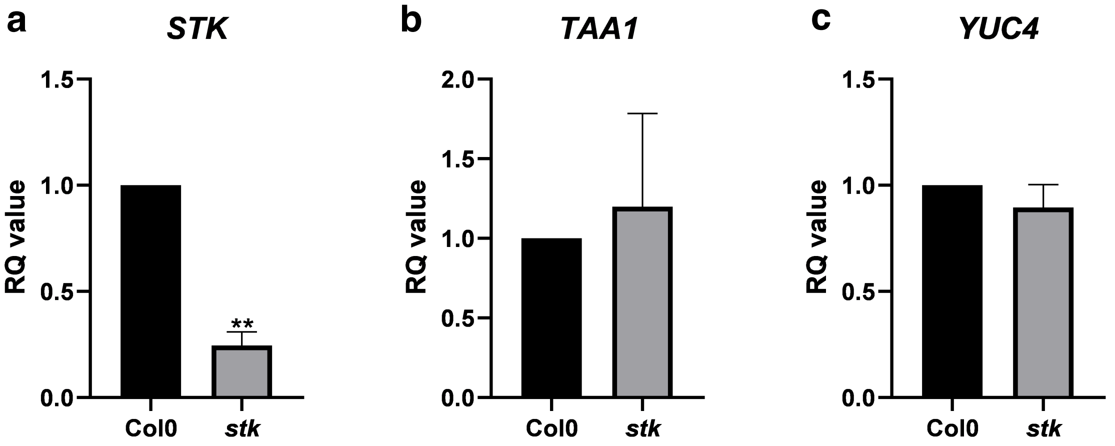

Supplementary Fig. S4 *TAA1* and *YUC4* expression is not regulated by STK

(a-c) The expression of *STK* **(a)** is reduced in *stk* gynoecium (stage 12), but not the expression of *TAA1* **(b)** and *YUC4* **(c)**. Relative quantification of transcript levels assessed by RT-qPCR in Col-0 and *stk* gynoecium. The values are represented as the means ± SD from three biological replicates. *p*-values are calculated with a two-tailed Student's *t*-test. **, $p < 0.01$.

Supplementary Table S1

**For real time PCR**	
AGL5	TCGAGTGTGATACATCAAGGGAC
	CGGTTCAAGAAGGTTAACCGC
TAA1	CGGTGGGAGAAGCTACGTGA
	CTTCGTCCCTAGCCACGC
YUC4	CGGATGGAAAGGAGAGAAGG
	CCTCAGCGATCTTAACGGCG
STK	TGGAGAGGTATCAACAACACC
	AACCTCCATTACCAGATCCAG
UBQ	GGCCTTGTATAATCCCTGATGAATAAG
	AAAGAGATAACAGGAACGGAAACATAGT
**For ChIP**	
**TAA1**	
P1	TGTCCATTGACAAAGCCTTT
	ATTTCTGATCATCTGAAGGAAC
P2	CATAGTCATCATTGAGCGG
	GTAAGGACGATTCAAGTCG
P3	CTACAAGATTTACTCTCGCC
	GGTGAGCTGTGGTATACTT
P4	CACTGACAATAAATGAAGCTG
	CTGCTCCGGTAAATTCAC
P5	TGCAGGAAAAGAAAACTCTAG
	GCTACCAAATGCGGAAAA
**YUC4**	
P1	CCAGCTTAGTCATGAGTCTAG
	CTTTAACTAGCACTACATAACCG
P2	CAGCTTAAACTACCATGCG
	GAGGAAGAGAAATATTGGGAC
P3	TCAGCTATTTAATCAGCCG
	TTATACTGGACGCAACAAC
P4	CGAGCATTTACACCACCTCA
	TCTCGGGAAAAACCAAGAAA
Actin	AACTTTCAACACTCCTGCCATG
	CTGCAAGGTCCAAACG
**For genotying**	
stk_f	CACTGTCCAAGAAATCAATGCCGC
stk_r	GGAACTCAAAGAGTCTCCCATCAG
shp1_f	GTGACGGAAGGAGGGTTGACG
shp1_r	GTCTACTGATGAGTTGTCACTAGG
shp2_f	GAGGATAGAGAACACTACGAATCGTC
shp2_r	CAGGTCAAGTCAATAGATTCCCTAC
TD_LB	GATGCACTCGAAATCAGCCAATTTTAGAC

Cloning

AGL5_CDS_F	TTGAAGACAAAATGGAGGGTGGTGCGAGTA
AGL5_CDS_R	TTGAAGACAACGAACCAACAAGTTGCAGAGGTGGTTG
proYUC4_F	GCGAAGCTTAGACCTACTCTACTCCCTCCA
proYUC4_R	CGCGGATCCGTCGACTAATAAAAGCGAAAGAG
proTAA1_F	GCGAAGCTTATCCTACAAGATTTACTCTCGC
proTAA1_R	CGCGGATCCTTCTCGCTACCAAATGCGGA
proAGL5_F	ttGAAGACaaGGAGTGTCACAGGGCTAGTAAAAG
proAGL5_R	ttGAAGACaaCATTTTCTATAAGCCCTAGCTGAAG
proAGLBpI_F	AAAGAAGACGTCTcCttTCTTTCTTTTTTCTTCTTTCTAAT
proAGLBpI_R	AAAGAAGACaaGgAGACAGTTGGGTTTGGATTGGG
