# Supplementary material for: The MADS-box protein SHATTERPROOF 2 regulates TAA1 expression in the gynoecium valve margins
Source: Plant Reprod. 2025 Jan 10;38(1):6. doi: 10.1007/s00497-024-00518-6 (PMC11723901; doi:10.1007/s00497-024-00518-6)
Supplement: Supplementary file 7 — Supplementary file7 (DOCX 15 KB) [file 497_2024_518_MOESM7_ESM.docx]

**Supplementary Figures and Tables**

**Supplementary Fig. S1** **Complementation of the phenotype of *shp1 shp2* mutant by *pSHP2: SHP2-GFP***

The Col-0 siliques (stage 17b) exhibit a well-developed dehiscence zone (dz). The dehiscence zone is absent in *shp1 shp2* mutant (Liljegren et al., 2000). The presence of *pSHP2: SHP2-GFP* restores the *shp1 shp2* mutant phenotype. (**a-c**) The complementation was analyzed by SEM on siliques of the same genotypes. (**d-i**) Lignification of the valve margin was assessed with phloroglucinol staining (**d-f**) and Safranin-O combined with Alcian Blue (**g-i**) on transversal sections at the base of the silique. The lignified cells marked by the dye are indicated by a black arrowhead. The genotypes are Col-0 (a, d, g), *shp1 shp2* (b, e, h) and *pSHP2: SHP2-GFP shp1 shp2* (c, f, i). Scale bars 100 μm.

**Supplementary Fig. S2 No obvious abaxial-adaxial defects in *shp1 shp2* gynoecium**

Cross-section of wild-type and *shp1 shp2* stage 12 gynoecium. Cells are marked by Toluidine blue. Scale bars: 100 μm.

**Supplementary Fig. S3 Close-up of *pTAA1:GFP-TAA1* expression pattern in stage 12 pistil**

*pTAA1:GFP-TAA1* expression in the cells at the valve-replum junction in wild-type gynoecium (stage 12). The fluorescent signal is green. Scale bar: 50 μm.

**Supplementary Fig. S4 *TAA1* and *YUC4* expression is not regulated by STK**

**(a-c)** The expression of *STK* **(a)** is reduced in *stk* gynoecium (stage 12), but not the expression of *TAA1* **(b)** and *YUC4* **(c)**. Relative quantification of transcript levels assessed by RT-qPCR in Col-0 and *stk* gynoecium. The values are represented as the means ± SD from three biological replicates. *p*-values are calculated with a two-tailed Student’s *t*-test. **, *p* <0.01.

**Supplementary Table S1** Primers used in the study.

**Supplementary Table S2** Detailed description of the identified proteins from IP MS analysis.

**Supplementary Video S1** *pTAA1:GFP-TAA1* expression in Col-0, video reconstruction from Z-stack.

**Supplementary Video S2** *pTAA1:GFP-TAA1* expression in *shp1 shp2*, video reconstruction from Z-stack.

**Supplementary Video S3** *pYUC4:3xnGFP* expression in Col-0, video reconstruction from Z-stack.

**Supplementary Video S4** *pYUC4:3xnGFP* expression in *shp1 shp2*, video reconstruction from Z-stack.
